# Leg blood flow and cardiac output are cyclically reduced during low‐intensity exercise with intermittent KAATSU cuff inflation in young adults

**DOI:** 10.14814/phy2.70551

**Published:** 2025-09-10

**Authors:** Stuart P. S. Mladen, Stacey P. A. Forbes, Abby K. Zedic, Patrick J. Drouin, Vaughn S. England, Michael E. Tschakovsky

**Affiliations:** ^1^ School of Kinesiology and Health Studies Queen's University Kingston Ontario Canada; ^2^ Department of Kinesiology McMaster University Hamilton Ontario Canada

**Keywords:** blood flow restriction, cardiac output, exercise, KAATSU, leg blood flow, oxygen delivery

## Abstract

The present investigation sought to determine the cardiovascular responses to a commercially available KAATSU cuff system with rhythmic cuff inflation–deflation periods during leg exercise. Seventeen participants performed two‐legged knee flexion/extension exercise at 25% of peak work rate (WR_Peak_) with bilateral KAATSU cuffs applied to the proximal thigh (KAATSU) or work‐rate matched control exercise (CTL). During KAATSU trials, the cuffs were set to Cycle Mode (repeated 30‐s inflation; 5‐s deflation) at progressively increasing cuff pressure (150–220 mmHg). Right leg blood flow (LBF; Doppler and echo ultrasound) and cardiac output (CO; finger photoplethysmography) were measured continuously. The deflated KAATSU cuffs impaired exercising LBF (*p* < 0.01), with no further impairment during the first cuff inflation (*p* > 0.99). Following the initial cuff inflation, deflated KAATSU cuffs no longer compromised LBF (*p* = 0.78). Subsequently, LBF (*p* < 0.01) and CO (*p* = 0.04) were compromised during each of the remaining cuff inflations, but the magnitude of compromise was not augmented by progressive increases in cuff pressure (2LBF interaction: *p* = 0.41; CO interaction: *p* = 0.40). KAATSU cuff inflation reduces exercising LBF to a similar extent across cuff pressures. Furthermore, reductions in exercising CO during cuff inflation were immediately restored upon deflation, revealing the dependency of CO on exercising leg perfusion and subsequent venous return.

## 1 INTRODUCTION

Exercise training “with additional pressure”—termed KAATSU Training—was established by Dr. Yoshiaki Sato in Japan, who developed the first KAATSU system for public use in 1983 (Sato, 2005). Dr. Sato's seminal work using KAATSU cuff systems (Sato, 2005; Takarada et al., 2000, 2002) has led to the upsurge in popularity of blood flow restriction (BFR) training, which involves the application of a pneumatic cuff or tourniquet to an exercising limb proximal to the muscles being trained with the intent to reduce muscle perfusion (Patterson et al., 2019; Scott et al., 2015). Performing low‐load resistance (Lixandrão et al., 2018; Slysz et al., 2016; Takarada et al., 2000) or aerobic (Abe et al., 2006; Ozaki et al., 2011) exercise training with BFR can enhance skeletal muscle adaptations when compared to non‐BFR training at an equivalent intensity and volume. Consequently, BFR has emerged as an alternative exercise training modality for individuals who are contraindicated to train under high‐loads.

Generally, external cuff inflation is proposed to cause exercising muscle hypoperfusion, resulting in impaired muscle oxygenation and increased metabolite accumulation, thereby augmenting the muscle metabolic disturbance at a given work rate (Pearson & Hussain, 2015). Quantifying the specific perturbations to flow imposed by external cuff inflation provides insight into the magnitude of the resultant metabolic disturbance, and ultimately the adaptations that would occur following repeated exercise training. Previous studies have evaluated cardiovascular responses to BFR leg exercise (Mladen et al., 2025; Singer et al., 2020). However, these studies have measured limb blood flow during a single restriction period at a fixed cuff pressure. The commercially available KAATSU cuffs, along with the manufacturer‐recommended Cycle mode protocol, fundamentally differ from previously measured BFR perturbations. First, KAATSU cuffs are more flexible and elastic, and significantly narrower (5 cm) than BFR cuffs (12 cm) that are used in research studies examining BFR training effects and cardiovascular responses. Second, the manufacturer‐recommended KAATSU Cycle mode protocol incorporates a series of intermittent cuff inflation and deflation periods with progressively increasing absolute cuff inflation pressures (KAATSU Canada, 2025). No previous studies have determined (1) the limb hemodynamic responses to cyclic cuff inflation/deflation periods, or (2) whether progressive increases in inflation pressure result in greater perturbations to exercising limb blood flow. Accordingly, the primary purpose of this study was to measure the peripheral cardiovascular responses to cyclic KAATSU cuff inflation/deflation during leg exercise. We tested the hypothesis that KAATSU, relative to control exercise, will impair exercising leg blood flow (LBF) during cuff inflation and this impairment will increase with cuff inflation pressure, but elevate LBF during cuff deflation due to the development of compensatory vasodilation, as we recently observed with sustained cuff inflation (Mladen et al., 2025).

Under conditions where external cuff application compromises exercising LBF, it is expected that total venous return would also be impaired. Given that cardiac output (CO) is ultimately limited to the rate of venous return (Magder et al., 2019), it is tempting to speculate that CO would also be reduced in exercise during the cuff inflation periods. However, several studies have demonstrated no difference in CO between BFR and non‐BFR exercise (Ozaki et al., 2010; Renzi et al., 2010; Staunton et al., 2015; Takano et al., 2005). The absence of a CO change with cuff inflation suggests that either (1) there is no cuff‐induced flow compromise during exercise or (2) increased flow in other vasculature offsets the impairment at the exercising legs. Furthermore, whether the effect of reduced and restored venous return from exercising muscle is rapid, such that CO changes rapidly with altered limb perfusion is also not known. Consequently, the secondary purpose of this study was to quantify the effects of cyclic KAATSU cuff inflation/deflation on total circuit flow. We tested the hypothesis that exercising CO would adjust in parallel with exercising LBF, such that reduced LBF would decrease venous return and subsequently CO.

## 2 METHODS

### 2.1 Participants and ethical approval

Seventeen young, healthy subjects (8F), (see Section 2.5 for [G*Power] power analysis description) participated in this study (see Table 1 in Results for participant characteristics). Participants attended an initial screening visit during which they completed a medical screening form and CSEP Get Active Questionnaire to assess study eligibility and physical activity readiness, respectively. Eligibility was assessed based on the following inclusion criteria: 18–30 years of age, nonsmoker. The exclusion criteria were as follows: presence of hypertension, cardiovascular disease, hypothyroidism, or use of medications that alter cardiovascular function. Eligible participants were provided a verbal and written explanation of the experimental protocol and its associated risks before providing informed written consent. Standard anthropometric data (age, height, and body mass) were obtained for each subject. Menstrual cycle phase was recorded for descriptive purposes, but not controlled for since exercising blood flow does not change across menstrual cycle phases (Weggen et al., 2023). All experimental procedures were approved by the Queen's University Health Sciences and Affiliated Teaching Hospitals Research Ethics Board (6005158 PHE‐036‐02), and conform to the standards set by the *Declaration of Helsinki*. To reduce outcome reporting bias, this study was registered through Open Science Framework (https://doi.org/10.17605/OSF.IO/5XBG7). All participant screening decisions were performed by a single experimenter. Participants were blinded to the study hypothesis for the duration of the study.

**TABLE 1:** Participant characteristics.

	Female, mean (*n* = 8)	Male, mean (*n* = 9)	Group, mean (*n* = 17)
Age (years)	23 ± 3	21 ± 1	22 ± 3
Height (cm)*	166 ± 6	183 ± 5	175 ± 10
Body mass (kg)*	60 ± 6	82 ± 11	71 ± 14
WR_Peak_ (W)*	93 ± 22	125 ± 20	110 ± 26
25% WR_Peak_ (W)*	23 ± 5	31 ± 5	27 ± 6
Follicular: Luteal (*n* = 7F)	4:3	—	—

*Note*: All values mean ± standard deviation.

Abbreviation: WR_Peak_, peak work rate.

* Significant (*p* < 0.05) difference between females and males.

### 2.2 Experimental design and conditions

This study utilized a randomized within‐subject repeated measures design, where participants completed five exercise tests over two sessions [one incremental ramp test to determine peak work rate (WR_Peak_) on day one and four 11‐min exercise protocols on day two]. Data collection took place between February 23rd, 2024 and July 12th, 2024 in the Human Vascular Control Lab at Queen's University in Kingston, Ontario, Canada. Participants were asked to avoid consuming large meals 4 h before, and caffeine and alcohol 12 h before the experimental session. Participants were also asked to abstain from strenuous lower body exercise for 48 h before both visits to the lab.

#### 2.2.1 Exercise modality

**FIGURE 1:**
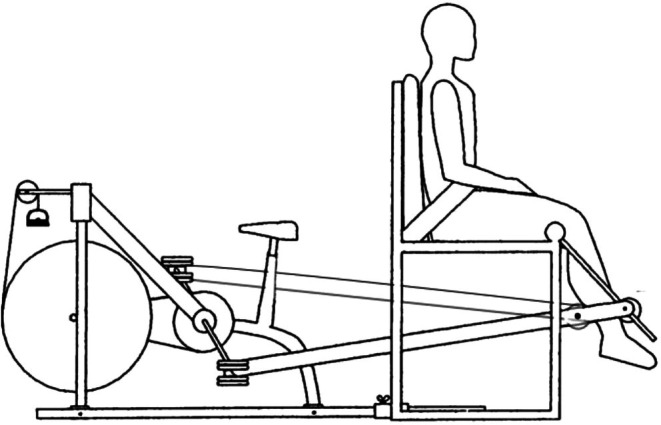
All exercise was performed on a two‐legged knee flexion/extension ergometer.

All exercise was performed on a two‐legged knee flexion/extension ergometer (Figure 1), as described previously (Mladen et al., 2025). Briefly, the flexion/extension ergometer allows for electrically braked work rate controlled exercise of the quadriceps and hamstring muscles in an alternating “kicking” motion. Importantly, the cycle ergometer can rapidly adjust its resistance to changes in revolutions per minute (RPM) to keep WR constant. However, to control for contraction frequency, participants maintained a cadence of 55 ± 3 cycles per leg per minute. To begin a trial, an experimenter manually cranked the ergometer flywheel 5 s before issuing a verbal command to the participants to begin exercise.

#### 2.2.2 Femoral artery diameter screening

At the start of the familiarization visit, participants were screened to ensure their common femoral artery (CFA) diameter exceeded 0.79 cm. CFA diameters that exceed 0.79 cm do not significantly dilate during exercise (Gonzales et al., 2010; Proctor & Parker, 2006). This allowed for a single CFA diameter measurement to be extrapolated throughout the test protocol for calculation of leg blood flow (LBF; see Section 2.4).

#### 2.2.3 Exercise modality familiarization

Next, participants performed three 5‐min bouts of flexion/extension exercise, separated by 2 min of rest. The intensities of each exercise bout were 0 W, 10 W, and 20 W, respectively. Participants were instructed to (1) exercise at 55 RPM, (2) completely relax knee extensors during knee flexion, and vice versa, and (3) keep hips as still as possible during the protocol. These cues were implemented to achieve optimal blood flow between extensor periods and reduce Doppler motion artifact (Shoemaker et al., 1994). These practice sessions acted to ensure that participants could properly coordinate leg flexion and extension exercise and that CFA blood velocity measurements could be accurately obtained during exercise.

#### 2.2.4 Establishing exercise work rates

At the end of the familiarization visit, each participant's WR_Peak_ was determined using a ramp protocol. The protocol began with 3 min of loadless two‐legged knee extension/flexion exercise at 55 RPM. This was followed by a 1 W increase in load every 5 s until volitional fatigue. The highest WR a participant achieved before volitional fatigue was selected as their WR_Peak_.

#### 2.2.5 Experimental visit and conditions

**FIGURE 2:**
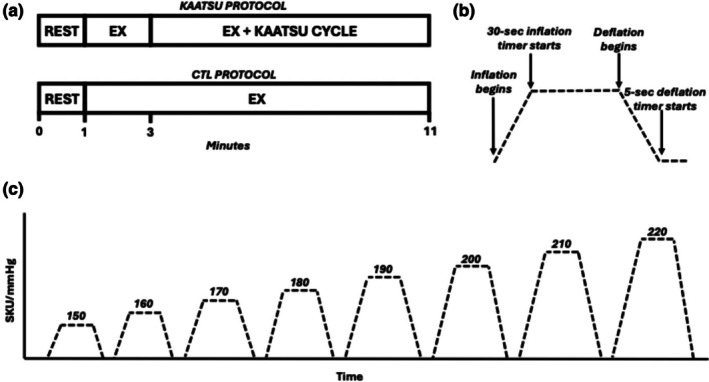
Schematic representation for (a) experimental visit test protocol, (b) timepoints within a single KAATSU Cycle, and (c) cuff pressure throughout the KAATSU Cycle protocol. For KAATSU Cycle, the time from the start of inflation to the desired pressure varied between participants. CTL, control; EX, exercise; SKU, standard KAATSU units.

On the experimental day, participants performed four, 11‐min test protocols (Figure 2). Both the control (CTL) and KAATSU conditions consisted of 1 min of rest, followed by 10 min of two‐legged flexion/extension exercise at 25% WR_Peak_ and 55 RPM. Prior to the KAATSU condition, pneumatic cuffs (width: 5 cm; KAATSU C3 AirBands; KAATSU Canada®, Thorold, Ontario, Canada) were applied to the most proximal portion of each leg. Participants were instructed to apply the KAATSU cuffs snugly, such that one, but not two fingers, could fit between the cuff and their leg. All participants had an upper leg circumference of >20 inches and were thus provided with the large‐sized KAATSU AirBand set. In the KAATSU conditions, KAATSU Cycle mode on the lowest setting was initiated following 2 min of exercise, which is a sufficient duration to reach steady state with this exercise modality (Mladen et al., 2025). KAATSU Cycle mode involves a 30‐s inflation period, followed by a 5‐s deflation period, through a series of progressively increasing pressures ranging from 150 to 220 Standard KAATSU Units (i.e., mmHg). The cuff pressure increased by 10 mmHg for each subsequent cuff inflation cycle (cycle 1: 150 mmHg, cycle 2: 160 mmHg, etc.). One set of KAATSU Cycle mode lasts approximately 7 min. However, this time is subject to variability between trials and participants as the 30‐s inflation and 5‐s deflation timers do not begin until the desired pressure is reached. The order of the trials was either (1) KAATSU, KAATSU, CTL, CTL, or (2) CTL, CTL, KAATSU, KAATSU, and this order was counterbalanced across participants. Each protocol was separated by ~10 min of quiet rest to allow CFA blood velocity to return to baseline levels.

### 2.3 Instrumentation and data acquisition

#### 2.3.1 Leg blood flow

We measured LBF at the CFA since (1) quality measurements are accessible during exercise and (2) it is the single conduit artery for flow into the legs and thus allows quantification of total LBF. All data collection was performed in a temperature‐controlled room (19–21°C) to minimize and stabilize blood flow to skin so that changes in CFA blood flow to the leg are indicative of muscle blood flow. CFA mean blood velocity (MBV) was measured in the right leg continuously during the exercise tests with a flat 4 MHz pulsed Doppler probe (model 500 V 131 Transcranial Doppler; Multigon Industries, Mt. Vernon, NY, USA) attached to the skin at a site ~3 cm proximal to the bifurcation of the CFA. CFA MBV data was collected (V) via an analog connection to a Powerlab/8sp (ADInstruments) and converted to cm/s in Labchart 7 software (ADInstruments) using a previously created calibration curve—this calibration accounts for the insonation angle of the Doppler ultrasound relative to the participants CFA. CFA diameter and angle relative to the skin surface was measured at the beginning and end, respectively, of each experimental visit with a linear echo ultrasound probe operating at 13 MHz in 2D mode (Vivid I; GE Medical Systems, London, ON, Canada), positioned at the same location as the Doppler probe. The single diameter measurement obtained at the beginning of the experimental visit was used for all LBF analysis. All LBF measurements were performed by the same investigator (S.M.).

#### 2.3.2 Central hemodynamics

A finger photoplethysmograph (Finometer MIDI; Finapres Medical Systems, Enschede, The Netherlands), placed on the participants left middle finger and kept at heart level, was used to measure systemic mean arterial pressure (MAP) and heart rate (HR). Stroke volume (SV) was calculated via the ModelFlow™ method using a nonlinear, 3‐element model of the aortic input impedance (Bogert & van Lieshout, 2005) taking height, body mass, age, and sex into account. CO was calculated from SV and HR.

### 2.4 Data analysis

#### 2.4.1 Leg blood flow

CFA diameters were quantified with measurements from Arterial Ultrasound Imaging (MAUI: Hedgehog Medical, https://hedgehogmedical.com/) automated edge detection software. LBF was calculated as MBV (cm/s) × 60 (s/min) × π × [femoral artery diameter (cm)/2]^2^. LBF values were doubled to represent blood flow to both exercising legs (two‐leg blood flow; 2LBF).

#### 2.4.2 Vascular conductance

**FIGURE 3:**
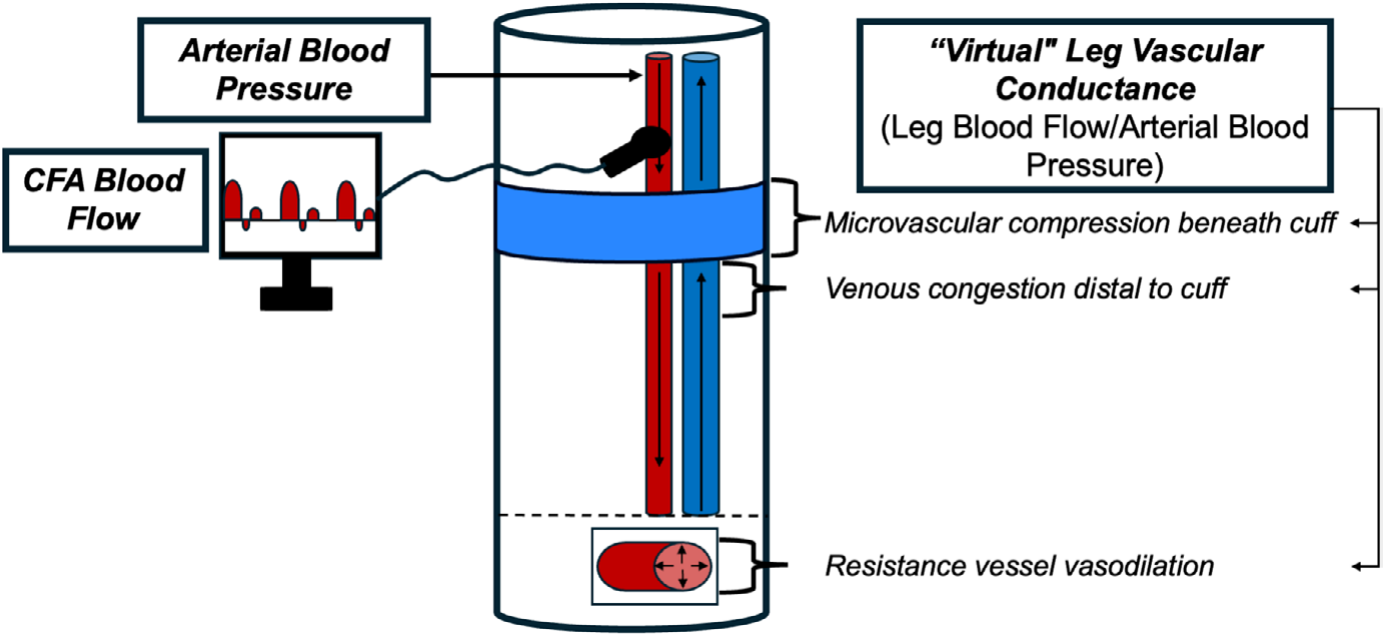
Schematic of leg blood flow, arterial blood pressure, and leg vascular conductance measurements. Doppler and echo ultrasound was used to measure common femoral artery (CFA) blood flow proximal to the KAATSU cuff, which reflects total perfusion through the exercising legs. Systemic arterial blood pressure—the driving pressure for leg blood flow—was measured via photoplethysmography. “Virtual” leg vascular conductance was calculated as leg blood flow/arterial blood pressure. Virtual vascular conductance encompasses (1) cuff compression force on microvasculature beneath the cuff, (2) venous congestion distal to cuff, (3) effect of muscle contractions on resistance vessel and venous compression, and (4) the vasodilation of resistance vessels beneath and distal to the cuff.

LBF through the CFA is determined by MAP (arterial driving pressure) and the combined effects of the BFR cuff compression, muscle contractions, and resistance vessel tone (Figure 3). Since these effects cannot be separated, their combined effect is quantified as a “virtual” leg vascular conductance (2LVC_VIRT_), calculated as 2LBF (mL/min)/MAP (mmHg). Total vascular conductance (TVC) was calculated as CO (mL/min)/MAP (mmHg).

#### 2.4.3 Measurement averaging for statistical comparisons

Resting measurements were averaged from −45 s to −15 s before exercise. Exercising measurements prior to cuff inflation were averaged from 60 s to 120 s of exercise. In the KAATSU trials, averages were taken for each measurement during the final 15 s of each cuff inflation period and the 5‐s deflation period. For each participant, the timepoints of each cuff inflation and deflation period were averaged, and the CTL averages were taken at the equivalent timepoints. Repeat trials of each condition were averaged to yield a single mean response for each participant. All exercising measurements were assigned to a cycle consisting of CTL, KAATSU Deflation (prior to cuff inflation), and KAATSU Inflation.

### 2.5 Statistical analysis

Descriptive statistics for baseline participant characteristics and experimental data were reported as mean ± SD. Paired *T*‐tests were run to test for differences between CTL and deflated KAATSU cuffs during rest. In our protocol, the first deflation period occurs prior to cuff inflation, and thus is not subject to any compensatory effects. Therefore, to determine the existence of potential compensatory effects following the first cuff inflation, we conducted two‐way repeated measures ANOVAs with condition (CTL, deflation, and inflation) and cycle (cycle 1 and cycle 2) as within‐subject factors for each hemodynamic variable. To evaluate whether there were differences beyond the initial cycles, additional two‐way repeated measures ANOVAs were run with condition (CTL, deflation, and inflation) and cycle (cycles 3–8; six within‐subject levels) as within‐subject factors. In the case of significant main or interaction effects, pairwise comparisons were performed using GraphPad Prism's Bonferroni‐adjusted post hoc tests. These included comparisons of condition within each cycle, and comparisons of cycles within each condition.

We found reductions in both 2LBF and CO during cuff inflation. We therefore conducted an exploratory two‐way repeated measures ANOVA with measurement (Δ2LBF, ΔCO) and cycle (cycles 1–8; eight within‐subject levels) as within‐subject factors to determine whether the peripheral flow reduction (Δ2LBF) was different from the systemic flow reduction (ΔCO). We also conducted a simple linear regression to determine whether Δ2LBF and ΔCO were correlated. *Cohen's d* values were calculated for *T*‐test comparisons, and values were interpreted as small (0.2), medium (0.5), and large (0.8) (Sullivan & Feinn, 2012). Partial eta‐squared ($\eta_{p}^{2}$) was calculated for the main and interaction effects of each two‐way repeated measures ANOVA and interpreted as small (≥0.01), medium (≥0.059), and large (≥0.138) (Cohen, 1988). All statistical testing was run using GraphPad Prism 10 software (GraphPad, San Diego, CA, USA). Statistical significance was accepted at *p* ≤ 0.05.

#### 2.5.1 Sample size calculation

An a priori sample size calculation was conducted for the primary outcomes: differences in LBF and CO between cuff pressure conditions. G*Power revealed that a sample size of 17 was required to detect a statistically significant 10% difference (medium effect size) in LBF for a repeated measures, between factors analysis (*f* = 0.383, *n* = 17; based on the means and variation found in MacDonald et al. (2000)) (α error probability = 0.05, 1−β error probability = 0.80). G*Power revealed that a sample size of 17 was required to detect a statistically significant 1.1 L/min difference (medium effect size) in CO for a repeated measures, between factors analysis (*f* = 0.379, *n* = 17; based on the means and variation found in (Ozaki et al., 2010)) (α error probability = 0.05, 1−β error probability = 0.80). Therefore, our target sample was 18 participants (9 females and 9 males).

## 3 RESULTS

### 3.1 Participant enrolment and completion

**FIGURE 4:**
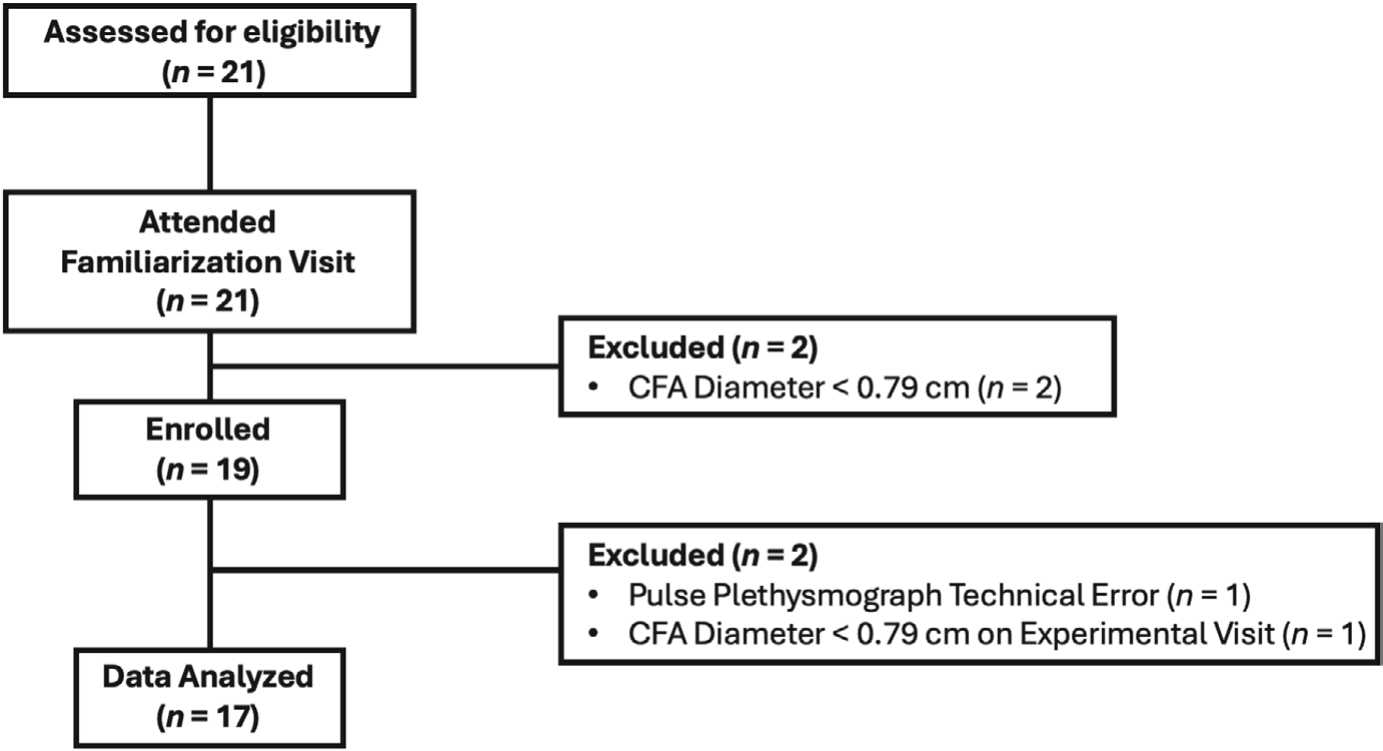
Consolidated Standards of Reporting Trials (CONSORT) participant flowchart. CFA, common femoral artery.

All 21 individuals assessed for eligibility (12F, 9 M) met the initial inclusion criteria and attended the familiarization visit (Figure 4). During the familiarization visit, two female participants had common femoral artery (CFA) diameters that were under the threshold (<0.79 cm) for inclusion. 19 (10F, 9M) participants were enrolled in the study. Due to a technical error with the pulse plethysmograph, one participant did not complete data collection. One participant's CFA diameter was under the threshold for inclusion on the day of the experimental visit and was excluded from final analysis. Thus, 17 (8F and 9M) participants were included in the final analysis.

### 3.2 Participant characteristics

Participant characteristics are presented in Table 1. Height, body mass, and WR_Peak_ were all significantly higher (*p* < 0.05) in males (Table 1). One female participant did not have regular menstrual periods and was not included in menstrual cycle phase reporting.

### 3.3 Did pre‐inflation KAATSU affect resting hemodynamics?

The group mean resting values are presented in Table 2. Paired *T*‐tests revealed no differences between conditions for any measurement.

**TABLE 2:** Resting values of CTL and pre‐inflation KAATSU (*n* = 17).

	CTL	KAATSU	Effect sizes
2LBF (mL/min)	640 ± 412	577 ± 241	Condition (*p* = 0.23, *d* = 0.19)
2LVC_VIRT_ (mL/min/mmHg)	8.9 ± 5.7	7.8 ± 3.3	Condition (*p* = 0.14, *d* = 0.24)
MAP (mmHg)	72.3 ± 11.1	74.4 ± 9.0	Condition (*p* = 0.39, *d* = 0.21)
CO (L/min)	5.6 ± 1.2	5.5 ± 1.2	Condition (*p* = 0.55, *d* = 0.08)
HR (beats/min)	70.6 ± 11.9	71.3 ± 11.6	Condition (*p* = 0.44, *d* = 0.06)
SV (mL)	80.7 ± 14.9	78.7 ± 16.8	Condition (*p* = 0.33, *d* = 0.13)
TVC (mL/min/mmHg)	79.2 ± 19.3	75.2 ± 18.5	Condition (*P* = 0.23, *d* = 0.21)

*Note*: All values mean ± standard deviation.

Abbreviations: 2LBF, two‐leg blood flow; 2LVC, two‐leg vascular conductance; CO, cardiac output; CTL, control; HR, heart rate; MAP, mean arterial pressure; SV, stroke volume; TVC, total vascular conductance.

### 3.4 Did KAATSU cycle mode alter exercising leg blood flow and its determinants?

#### 3.4.1 Initial onset of KAATSU inflation (cycles 1 versus 2)

**FIGURE 5:**
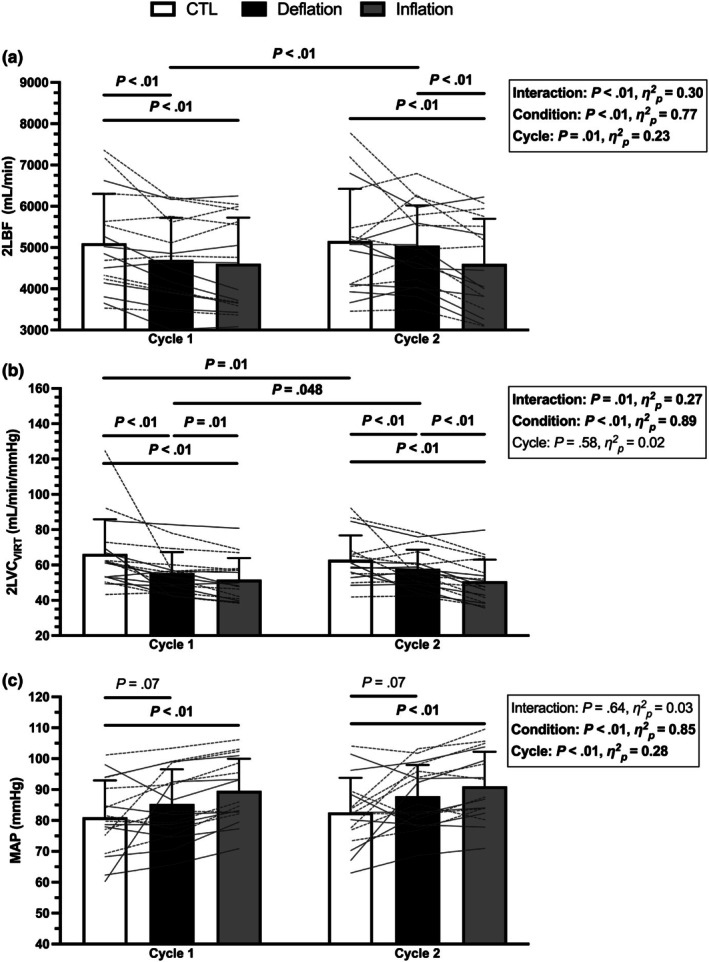
The effect of the first two KAATSU cycles (150–160 mmHg) on (a) two‐leg blood flow (2LBF), (b) virtual two‐leg vascular conductance (2LVC_VIRT_), and (c) mean arterial pressure (MAP). Individual participant data are presented as the thin horizontal/angled gray lines, with male participants represented by dashed lines, and females represented by solid lines. Significance indicated by *p* < 0.05.

2LBF, 2LVC_VIRT_, and MAP values across the first two cuff inflation cycles are presented in Figure 5. There was a significant condition × cycle interaction effect for 2LBF (*p* < 0.01). Prior to the first cuff inflation, the deflated KAATSU cuffs reduced exercising 2LBF compared to CTL (*p* < 0.01). The first cuff inflation to 150 mmHg did not augment the 2LBF impairment compared to deflation (*p* > 0.99). After the first cuff inflation, the deflated KAATSU cuffs no longer impaired 2LBF vs. CTL (*p* = 0.78). The second cuff inflation to 160 mmHg reduced 2LBF compared to both CTL (*p* < 0.01) and the preceding cuff deflation period (*p* < 0.01). 2LBF during deflation increased from cycle 1 to cycle 2 (*p* < 0.01).

There was a significant condition × cycle interaction effect for 2LVC_VIRT_ (*p* < 0.01). 2LVC_VIRT_ was impaired prior to the initial cuff inflation by the deflated KAATSU cuffs (*p* < 0.01). Cuff inflation to 150 mmHg further blunted 2LVC_VIRT_ (*p* = 0.02). Following the first cuff inflation, the deflated cuffs impaired 2LVC_VIRT_ (*p* < 0.01). Cuff inflation to 160 mmHg reduced 2LVC_VIRT_ compared to both CTL (*p* < 0.01) and cuff deflation (*p* < 0.01). 2LVC_VIRT_ during CTL decreased from cycle 1 to cycle 2 (*p* < 0.01). 2LVC_VIRT_ during deflation increased from cycle 1 to cycle 2 (*p* = 0.048).

There was a significant main effect of condition for MAP (*p* < 0.01). MAP was increased during cuff inflation compared to CTL in cycle 1 and cycle 2 (*p* < 0.01). MAP during cuff deflation approached a significant increase (*p* = 0.07).

#### 3.4.2 KAATSU cycles 3–8

**FIGURE 6:**
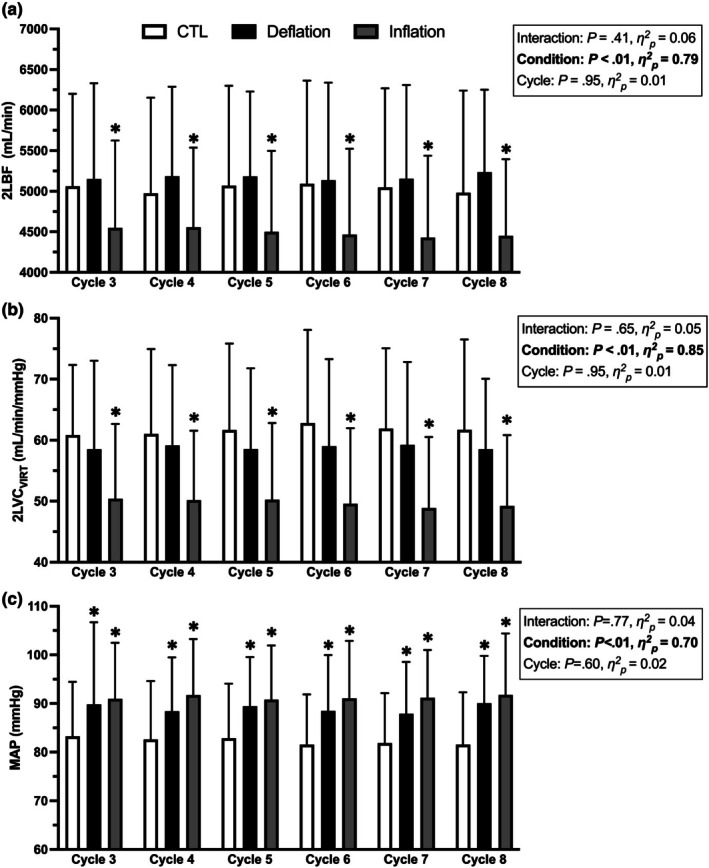
The effect of KAATSU Cycles 3–8 (170–220 mmHg) on (a) two‐legged blood flow (2LBF), (b) virtual two‐leg vascular conductance (2LVC_VIRT_), and (c) mean arterial pressure (MAP). *Significant difference from CTL (*p* < 0.05).

2LBF, 2LVC_VIRT_, and MAP values across cycles 3–8 are presented in Figure 6. There was a main effect of condition for 2LBF (*p* < 0.01), 2LVC_VIRT_ (*p* < 0.01), and MAP (*p* < 0.01). KAATSU cuff inflation significantly reduced exercising 2LBF compared to CTL (*p* < 0.01) and cuff deflation (*p* < 0.01). Similarly, 2LVC_VIRT_ was impaired by cuff inflation compared to CTL (*p* < 0.01) and cuff deflation (*p* < 0.01). MAP was elevated during KAATSU for both cuff inflation (*p* < 0.01) and cuff deflation (*p* = 0.01) compared to CTL. MAP was not significantly different between cuff inflation and deflation (*p* = 0.99).

### 3.5 Did KAATSU cycle mode alter exercising cardiac output and its determinants?

#### 3.5.1 Initial onset of KAATSU inflation (cycles 1 versus 2)

**FIGURE 7:**
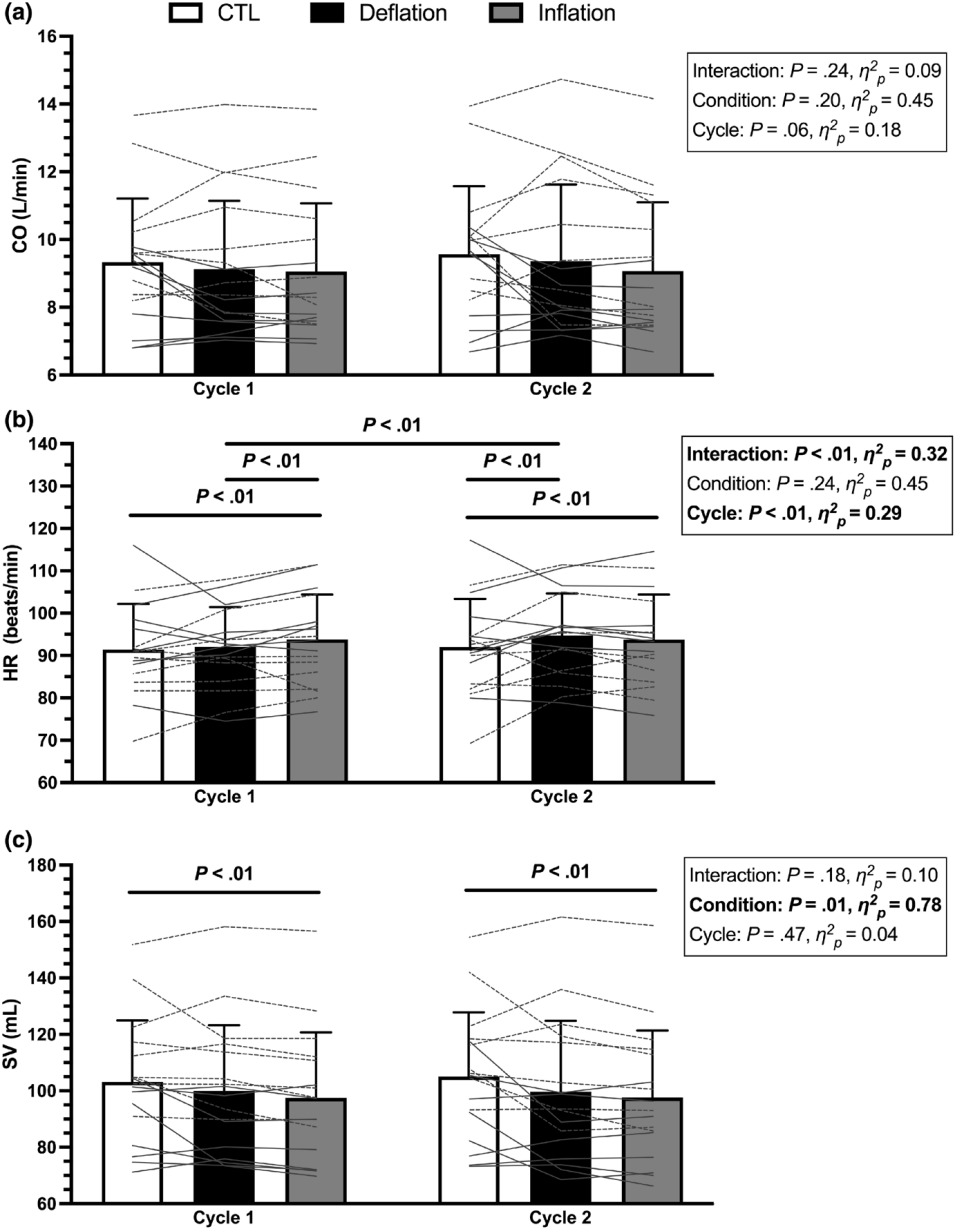
The effect of the first two KAATSU cycles (150–160 mmHg) on (a) cardiac output (CO), (b) heart rate (HR), and (c) stroke volume (SV). Individual participant data are presented as the thin horizontal/angled gray lines, with male participants represented by dashed lines, and females represented by solid lines. Significance indicated by *p* < 0.05.

CO, HR, and SV values across the first two cuff inflation cycles are presented in Figure 7. There were no main or interaction effects for CO. A significant condition × cycle interaction effect was observed for HR (*p* < 0.01). The first cuff inflation to 150 mmHg increased HR compared to CTL (*p* < 0.01) and deflation (*p* = 0.01). Upon cuff deflation, HR was still elevated compared to CTL (*p* < 0.01). The second cuff inflation to 160 mmHg did not augment the increased HR (*p* = 0.41). HR during cuff deflation increased from cycle 1 to cycle 2 (*p* < 0.01). There was a significant main effect of condition for SV (*p* = 0.01). SV was blunted during both cuff inflation periods compared to CTL (*p* = 0.01).

#### 3.5.2 KAATSU cycles 3–8

**FIGURE 8:**
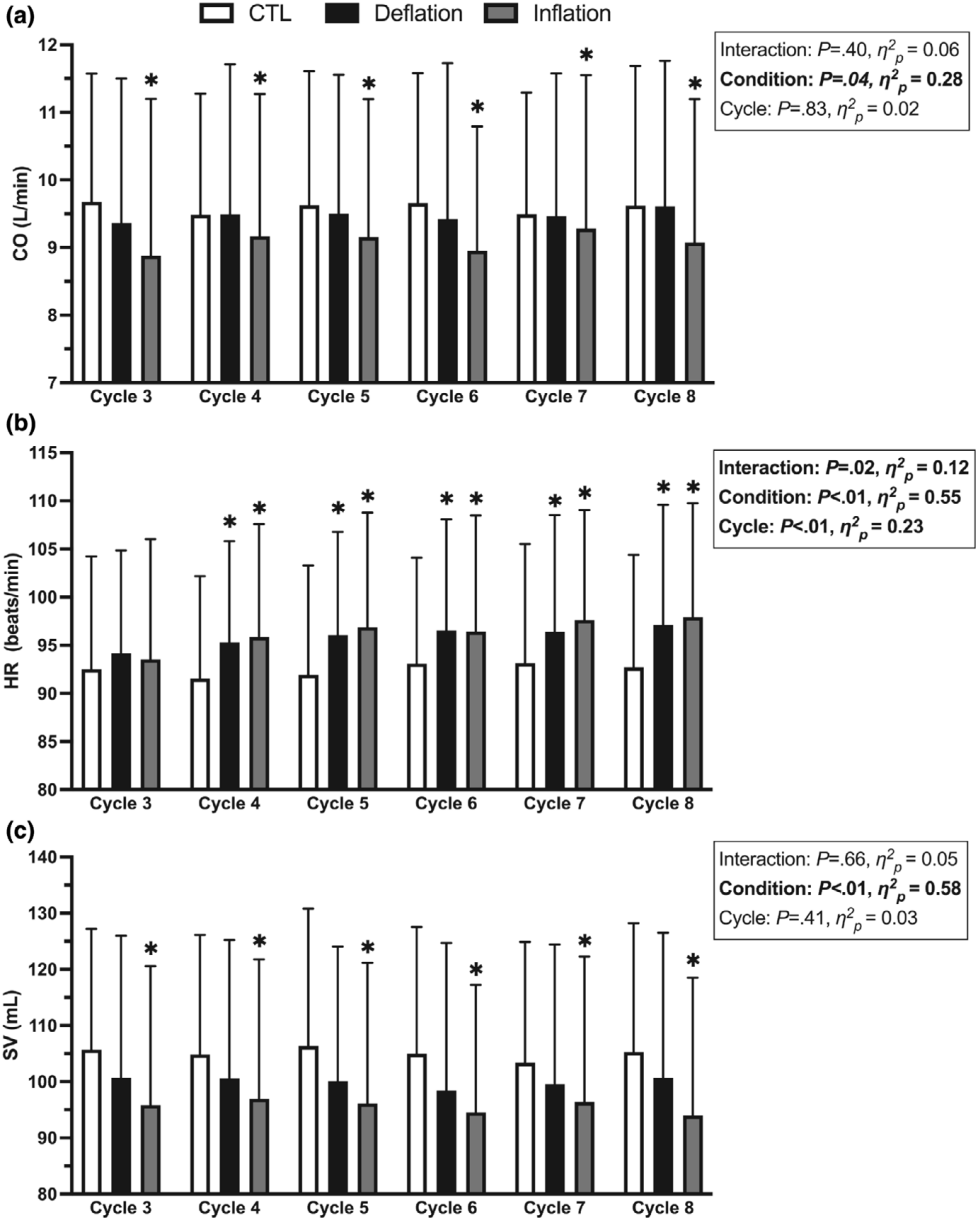
The effect of KAATSU Cycles 3–8 (170–220 mmHg) on (a) cardiac output (CO), (b) heart rate (HR), and (c) stroke volume (SV). *Significant difference from CTL (*p* < 0.05).

CO, HR, and SV values across the cycles 3–8 are presented in Figure 8. There was a main effect of condition for CO (*p* = 0.04). KAATSU cuff inflation significantly reduced exercising CO compared to CTL (*p* = 0.045). There was a significant condition × cycle interaction effect for HR (*p* = 0.02). There were no HR differences between conditions during cycle 3. During cycles 4–8, KAATSU cuff inflation and deflation increased HR compared to CTL (all *p* < 0.01). There was a main effect of condition for SV (*p* < 0.01). KAATSU cuff inflation significantly reduced exercising SV compared to CTL (*p* < 0.01).

### 3.6 Was the peripheral flow reduction related to the central flow reduction?

The Δ2LBF (579 ± 222 mL/min) was larger than the ΔCO (336 ± 159 mL/min) (KAATSU Deflation‐Inflation; Interaction: *p* = 0.44, $\eta_{p}^{2}$ = 0.06; Condition: *p* < 0.01, $\eta_{p}^{2}$ = 0.14; Cycle: *p* < 0.01, $\eta_{p}^{2}$ = 0.23). However, linear regression analysis showed a significant correlation between Δ2LBF and ΔCO (*R* ^2^ = 0.14, *p* < 0.001).

## 4 DISCUSSION

The purpose of this study was to test the following hypotheses: Compared to CTL (1) KAATSU will impair exercising LBF during cuff inflation and this impairment will increase with cuff inflation pressure, (2) KAATSU will result in higher exercising LBF during cuff deflation, and (3) exercising CO will follow changes in exercising LBF. The primary findings of this study were as follows: (1) applied but deflated KAATSU cuffs impair exercising LBF, (2) the first cuff inflation did not further compromise LBF, while all subsequent inflations compromised blood flow versus deflation and CTL, (3) increasing cuff inflation pressures did not augment the magnitude of perfusion compromise, and (4) CO was rapidly coupled to changes in LBF, except for during the first incidence where cuff inflation impaired perfusion.

### 4.1 Peripheral cardiovascular responses to KAATSU cycle

Reductions in LBF with the application of KAATSU have been observed previously during rest (Iida et al., 2007; Takano et al., 2005). Recent studies have also evaluated BFR cuff‐mediated flow reductions during handgrip exercise (Kilgas et al., 2019) and leg exercise (Mladen et al., 2025; Singer et al., 2020). However, these exercising studies have only evaluated BFR cuffs between 10 and 14 cm in width applied with a constant pressure (Kilgas et al., 2019; Mladen et al., 2025; Singer et al., 2020). Such characteristic differences between traditional BFR and the smaller, 5 cm KAATSU cuffs (Iida et al., 2007; Takano et al., 2005) may impose differential disturbances to the underlying exercising muscle vasculature. Given that the magnitude of hypoperfusion influences the metabolic disturbance and associated muscle adaptations from KAATSU and/or BFR, further investigation into the specific cardiovascular disturbances and responses imposed from exercise training with cuff‐inflation mediated hypoperfusion is warranted.

In our study, participants were instructed, as per manufacturer instructions, to snugly apply the KAATSU AirBands to “one‐finger tightness” prior to the KAATSU trials. KAATSU Cycle mode was initiated following 2 min of steady state exercise. Cycle mode consists of the KAATSU AirBands intermittently inflated for short periods (30 s) at progressively increasing pressures from 150 to 220 mmHg, with deflation periods of 5 s in between. During the initial 1‐min rest period, there were no differences in any variable between KAATSU and CTL. However, during the first 2 min of exercise, the deflated KAATSU AirBands applied as per manufacturer instructions were sufficient to reduce exercising LBF and 2LVC_VIRT_. KAATSU cuffs have greater elasticity compared to most traditional BFR cuffs, which are nylon and have limited stretch. Due to this characteristic difference, KAATSU cuffs can be applied snugly. It appears that the recommended snugness of the cuffs can compromise flow even when deflated. On further note, MAP during this initial cuff deflation approached significance (*p* = 0.07), indicating that some pressor compensation may have been present.

Interestingly, the first KAATSU cuff inflation to 150 mmHg did not cause a further reduction in LBF compared to the preceding deflation period. Although the first inflation compromised 2LVC_VIRT_, this was compensated for by an increased MAP, which increases the arterio‐venous pressure gradient for LBF. This finding aligns with several other studies observing an elevated MAP response during exercise with cuff inflation (Mladen et al., 2025; Renzi et al., 2010; Staunton et al., 2015; Thomas et al., 2018), likely caused by stimulation of the exercise pressor reflex via metabolite accumulation (Drouin et al., 2023; Grotle et al., 2020). Upon release of the first cuff inflation, the deflated cuffs no longer impaired LBF compared to CTL, and the magnitude of impairment to 2LVC_VIRT_ by the deflated cuffs decreased across the first two cycles. However, the magnitude of the MAP response to cuff deflation did not change across cycle 1 versus 2. We hypothesized that flow would be higher during cuff deflation compared to CTL due to compensatory vasodilation. However, since the initial deflation period reduced flow versus CTL, while the second deflation was not different from CTL, these findings may still reflect compensatory vasodilation triggered by the initial cuff inflation. Previous observations from our laboratory showing a compensatory vasodilation response to BFR under constant cuff pressure occur between 15 s and 1 min of exercise (Mladen et al., 2025). Compensatory vasodilation has also been observed in other models following a disturbance to oxygen delivery to demand matching (Bentley et al., 2011, 2014; Casey & Joyner, 2009), with evidence suggesting a key role for nitric oxide in mediating these effects (Bentley et al., 2017; Casey et al., 2010). Alternatively, it may be that the first cuff inflation stretches the cuff, reducing its ability to impede flow during subsequent deflations. Future studies should determine whether higher initial cuff pressures can further reduce limb perfusion compared to deflated cuffs, and whether similar compensatory responses exist at these higher pressures.

During cycles 3–8, KAATSU inflation reduced LBF and 2LVC_VIRT_ compared to CTL, while cuff deflation had no effect relative to CTL. These findings are consistent with those observed in cycle 2, which indicate that the absence of flow restriction by the deflated cuffs may be due to compensation initiated by the preceding cuff inflation period, or, alternatively, mechanical changes in the cuff (i.e., stretching) which limit perfusion compromise. For all cycles, MAP was higher during cuff inflation versus CTL and remained elevated above CTL during the 3rd to 8th cuff deflation periods. However, the magnitude of the MAP elevation during deflation was not of sufficient magnitude (~5–10 mmHg) to significantly augment perfusion compared to CTL. Nonetheless, the sustained elevation in MAP during deflation suggests that the MAP increases by KAATSU are due to exercise pressor reflex signaling (Grotle et al., 2020), rather than cuff‐induced reductions in outflow from the arterial circulation.

Two putative candidates may underlie the cuff‐induced compromises in LBF. First, there is microvasculature directly underneath the KAATSU cuffs that is subject to direct compression. That said, the contribution of a direct microvascular impairment may be trivial due to the relatively small width of KAATSU cuffs (5 cm). Second, the cuff may also impair venous outflow from the exercising limb, leading to venous congestion. This congestion effect would increase venous pressure, thereby decreasing the arteriovenous gradient for blood flow. In the context of exercise, it is important to acknowledge that a cuff‐mediated flow impairment may be offset by mechanical characteristics of muscle contraction. If muscle contractions increase limb venous pressure above the KAATSU cuff pressure, it would expel venous volume past the cuff, potentially eliminating or reducing the venous congestion effect.

### 4.2 Implications for BFR/KAATSU training

It is commonly assumed that higher cuff pressures will always lead to greater reductions in exercising limb blood flow in a dose–response manner. Contrastingly, the present study showed that progressively increasing cuff pressure throughout KAATSU Cycle mode did not lead to further reductions in leg perfusion compared to CTL. Similarly, a study demonstrated that resting flow is impaired nonlinearly with increasing cuff pressure (Mouser et al., 2018). The extent of hypoperfusion in the exercising limb modulates the local metabolic disturbances and, consequently, influences the muscle adaptations that follow a period of KAATSU or BFR training. Therefore, we speculate that muscle adaptations to KAATSU training are unlikely to be altered by increasing cuff pressure within the ranges included in this study. Supporting this, training adaptations to BFR are not increased with cuff pressures between 40% and 80% arterial occlusion pressure (Counts et al., 2016; Lixandrão et al., 2018). Further studies should investigate the leg perfusion responses to KAATSU over a broader range of cuff pressures to determine whether distinct “thresholds” of pressure exist, beyond which additional impairments to flow are observed.

This was the first study to date evaluating the magnitude of LBF compromise during exercise with KAATSU cuffs. The magnitude of the CFA flow compromise in the current study was approximately 10%, which is substantially lower than the LBF reductions (~30%–40%) observed in studies using larger BFR cuffs (Mladen et al., 2025; Singer et al., 2020). Specifically, our previous study, which used 14 cm wide cuffs inflated to 40% AOP, initially impaired flow by ~37%. Interestingly, the greater flow compromise seen in Mladen et al. (2025) is not due to differences in cuff pressure per se, since the average absolute pressure of 40% AOP in that study was ~73 mmHg. Rather, wider cuffs require a lower absolute pressure to occlude resting flow (Loenneke et al., 2012), and induce direct microvascular compression over a larger surface area. These findings highlight the importance of understanding the differential impact of cuff type in achieving a specific magnitude of limb blood flow compromise, which is relevant in a training context.

### 4.3 Circuit flow dynamics during KAATSU cycle

Aerobic exercise with cuff inflation is typically characterized by an increased HR and lower SV such that CO is unchanged compared to non‐BFR exercise (Ozaki et al., 2010; Renzi et al., 2010; Staunton et al., 2015; Sugawara et al., 2015; Takano et al., 2005). The reduction in stroke volume is often interpreted as a cuff‐mediated reduction in venous return, which is “compensated” for by an increase in HR (Cognetti et al., 2022; Iida et al., 2007; Ozaki et al., 2010; Renzi et al., 2010; Takano et al., 2005). However, this interpretation is problematic because a reduction in total venous return would manifest as a reduction in CO (Magder et al., 2019), which had not been observed in BFR exercise. Additionally, increases in HR may not actually compensate for reductions in SV if the rate of return of blood to the heart is decreased or unchanged (Bada et al., 2012). Indeed, progressive increases in HR (via atrial pacing) result in a proportional decrease in stroke volume, such that cardiac output is unchanged (Bada et al., 2012), which is in support of a peripherocentric determination of circuit flow (Drouin et al., 2025).

Recent observations from our laboratory found that CO is transiently reduced at the beginning of exercise with BFR, returning to unchanged levels by steady state (Mladen et al., 2025). In the present study, CO was not reduced by the first two cuff inflation periods. However, during cycles 3–8 we observed consistent reductions in CO during each 30‐s cuff inflation period, and no differences during the cuff deflation periods. These inflation‐dependent CO reductions are not afterload mediated since MAP was elevated equally during both cuff inflation and deflation. Rather, the findings from cycles 3 to 8 suggest that a cuff‐mediated LBF reduction initially decreases total venous return to the heart, which manifests as a reduction in CO (Magder et al., 2019). Under conditions of prolonged cuff‐impairment, the baroreflex may respond to an increase in MAP by lowering sympathetic outflow to the non‐exercising vasculature, resulting in vasodilation (Sheriff et al., 1990). This would increase peripheral blood flow and therefore venous return from other tissues, abrogating the cardiac output reduction by steady state exercise, as is typically observed (Ozaki et al., 2010; Renzi et al., 2010; Staunton et al., 2015; Sugawara et al., 2015; Takano et al., 2005).

Notably, following the first cuff inflation in our study, HR was not different between KAATSU cuff inflation and deflation. The fact that the HR dynamics commonly observed during BFR remain despite a restoration of circuit flow during cuff deflation suggests that these alterations are a product of the pressor response, which aligns with our MAP data. However, SV was inflation‐dependent such that it was lower during inflation compared to CTL, but it was not reduced during deflation. Therefore, the reduced CO during KAATSU inflation, but not deflation, is a function of cardiac filling alterations that reflect venous return coupled with the fluctuations in LBF. Together, our data demonstrate rapid temporal coupling of exercising limb blood flow alterations with CO changes, which is consistent with peripheral flow (venous return) driving changes in CO.

### 4.4 Methodological considerations

The utilization of separate ultrasound probes for vessel diameter and velocity measurements is the standard approach within our lab (Drouin et al., 2022; Mladen et al., 2025), given the limitations of the 13 MHz echo ultrasound probe for velocity assessment. The Multigon Doppler probe insonates a larger cross‐section of the vessel and captures the slower blood velocities near the vessel wall, thereby reducing the risk of flow overestimation. In the present study, the seated posture of participants and the proximal placement of the KAATSU cuffs limited the space to feasibly record with the echo and Doppler probe simultaneously. We therefore measured common femoral artery (CFA) diameter once at the beginning of the experimental visit, and this diameter was used for exercising LBF calculations. Evidence shows that the CFA does not dilate during exercise given that an individual's CFA diameter is greater than 0.79 cm (Gonzales et al., 2010; Proctor & Parker, 2006), and we excluded participants (*n* = 2) who were under this threshold. In the hypothetical scenario where CFA diameter did change from rest to exercise, the absolute magnitude of LBF for a given individual would be higher than calculated. However, any CFA diameter changes would be established by steady‐state exercise, and the first KAATSU cuff inflation was not initiated until steady‐state exercise was reached. Therefore, potential changes to CFA diameter would not impact identification of minor, rapid alterations in LBF during intermittent cuff inflation/deflation. Importantly, the lack of continuous diameter measurements is inconsequential to the interpretation of our LBF data as intermittent cuff inflation altered flow instantaneously, which is inexplainable by CFA dilation changes. Indeed, for the direction of change in mean blood velocity to be explained by CFA diameter changes, immediate CFA constriction with cuff release and CFA dilation with cuff inflation would have to occur, which is not consistent with the mechanisms of flow‐mediated dilation.

The ModelFlow method of CO measurement has not been validated for exercise with external cuff compression per se. However, the ModelFlow has been validated for producing reliable measures of CO change in exercise, during which muscle contractions compress the vasculature, alter flow patterns, and rapid changes in peripheral resistance occur (Stok et al., 1993; Sugawara et al., 2003; Wesseling et al., 1993). Furthermore, the ModelFlow accurately measures changes in CO induced by lower body negative pressure where significant shifts in blood volume distribution occur (Reisner et al., 2011; Shibasaki et al., 2011).

Aiming to address the underrepresentation of female participants in BFR research (Counts et al., 2018) and exercise science more broadly (Costello et al., 2014), we recruited a balanced number of young healthy male (*n* = 9) and female (*n* = 8) participants. While pooling the sexes provided adequate power to address our primary research question, we did not examine sex‐specific responses, and acknowledge that sex differences may have resulted in greater variability within the dataset. We recommend that future studies conduct adequately powered comparisons to account for potential sex differences in the hemodynamic responses to exercise with external cuff inflation.

Finally, although the flexion/extension ergometer allowed for quality LBF measurements during exercise, the results obtained may not be transferable to more traditional exercise modalities (e.g., cycling).

## 5 CONCLUSION

The present study found that commercially available KAATSU cuffs set to 150–220 mmHg reduce exercising LBF, but to a smaller magnitude compared to previous BFR studies. Moreover, the KAATSU Cycle protocol does not induce progressively greater flow impairments with increasing inflation pressures, supporting a nonlinear relationship between cuff pressure and flow compromise. When applied to a training context, higher cuff pressures may not always further augment muscle adaptations. Furthermore, CO was reduced during cuff inflation and quickly restored during deflation, suggesting that altering venous return from the exercising limb can quickly and repeatedly affect CO. Future research should explore the relationship between cuff pressure and perfusion impairment across a broader range of KAATSU cuff pressures.

## AUTHOR CONTRIBUTIONS

SPSM and MET were involved in conceptualization. SPSM, SPAF, AKZ, VSE, and PJD were involved in data curation. SPSM was involved in formal analysis, validation, visualization, and writing—original draft. SPSM, SPAF, AKZ, VSE, PJD, and MET were involved in investigation, methodology, project administration, and writing—review and editing. MET was involved in resources and software.

## FUNDING INFORMATION

Support for this project was provided by the Natural Sciences and Engineering Research Council (NSERC) of Canada Discovery Grant RGPIN‐2022‐05309 and Research Tools and Instruments Grant EQPEQ0407690‐11, as well as infrastructure grants from the Canadian Foundation for Innovation and the Ontario Innovation Trust (to M.E. Tschakovsky). S.P.S.M. was funded by an Ontario Graduate Scholarship.

## CONFLICT OF INTEREST STATEMENT

The authors declare there are no competing interests.

## Data Availability

Data generated or analyzed during this study are available from the corresponding author upon reasonable request.
